# Neonatal central nervous system infection by *Ureaplasma* species is rare, but relevant: results from a multicenter nationwide surveillance study

**DOI:** 10.1007/s15010-024-02435-2

**Published:** 2024-11-25

**Authors:** Christine Silwedel, Sarah V. Schnee, Johannes Liese, Birgit Henrich, Christoph Härtel, Christian P. Speer, Kirsten Glaser

**Affiliations:** 1 University Hospital Würzburg, University of Würzburg, Department of Pediatrics, Josef-Schneider-Str 2, 97080 Würzburg, Germany; 2 Institute of Medical Microbiology and Hospital Hygiene, University Clinic of Heinrich-Heine University Düsseldorf Universitütsstr 1, 40225 Düsseldorf, Germany; 3 Division of Neonatology, Department of Women’s and Children’s Health, University of Leipzig Medical Center, Liebigstraße 20a, 04103 Leipzig, Germany

**Keywords:** Meningitis, *Ureaplasma urealyticum*, *Ureaplasma parvum*, Preterm infant, Neonate, Neuroinflammation

## Abstract

**Purpose:**

*Ureaplasma species* (spp.) are relevant contributors to preterm birth but may also cause invasive infections particularly in very immature preterm infants. This study aimed to assess the incidence of neonatal *Ureaplasma* infections of the central nervous system (CNS).

**Methods:**

A nationwide epidemiological study on *Ureaplasma* CNS infection in preterm and term neonates and infants below 12 months of age was conducted between 10/2019 and 09/2022, using the German Pediatric Surveillance Unit (ESPED).

**Results:**

Over a period of three years, five infants with *Ureaplasma* meningitis were reported, amended by three cases treated in our own hospital. All infants had a history of extreme preterm birth. Clinical presentation varied and included non-specific neurological symptoms, such as developmental delay, in some cases, and acute, sepsis-like conditions in others, with increased apneas, irritability, and seizures. As the most consistent finding, internal hydrocephalus was reported, paralleled by pathological cerebrospinal fluid assessment characterized by mild pleocytosis, persistently elevated protein levels, and remarkably low or undetectable glucose levels, prompting further diagnostics. Treatment protocols varied and included off-label regimens. *Ureaplasma* eradication was verified by negative CSF-PCR and/or culture in most cases. Despite successful eradication, long-term neurological impairment was present in all infants.

**Conclusion:**

Although seemingly rare, *Ureaplasma*-driven neuroinflammation relevantly contributes to long-term morbidity of affected preterm infants.

## Introduction

Throughout European countries, about 10% of all infants are born prematurely [1]. Given its significant impact on neonatal outcome, preterm birth is a major medical challenge [2]. Amniotic infection and chorioamnionitis are key contributors to preterm birth, with *Ureaplasma (U.)* species (spp.) being among the pathogens most frequently involved [3, 4]. *U. urealyticum* and *U. parvum* commonly colonize the adult urogenital tract and can be detected in up to 80% of pregnant women [5]. While often regarded as non-virulent, *Ureaplasma* spp. can cause ascending infection during pregnancy and thus contribute to preterm birth [5]. Vertical or horizontal pathogen transmission seems to occur frequently and may result in fetal and/or neonatal *Ureaplasma* colonization or infection, with the most immature infants bearing the highest risk [3, 5, 6]. *Ureaplasma* spp. have been identified as causative agents in pneumonia and neonatal sepsis [5, 7]. Furthermore, case reports have described neonatal central nervous system (CNS) infection caused by *Ureaplasma* spp [8–12]. While clinical data are sparse, recent in vitro and animal studies have demonstrated a distinct neuroinflammatory capacity of *Ureaplasma* spp., suggesting an underestimated relevance of these pathogens in neonatal CNS inflammation [13–18]. This study aimed to systematically assess the incidence and clinical relevance of *Ureaplasma*-driven neonatal CNS infection. We discuss our findings against the background of existing literature and point to potential underlying mechanisms reported in experimental and animal studies.

## Methods

### Study design and data collection

This prospective study was conducted as part of the German Pediatric Surveillance Unit (*Erhebungseinheit für Seltene Pädiatrische Erkrankungen in Deutschland*, ESPED). ESPED is the official surveillance unit of the German Society of Pediatric and Adolescent Medicine, providing the infrastructure to conduct nationwide surveillance studies (www.unimedizin-mainz.de/esped). Reporting to the ESPED is voluntary, but highly accepted, with the majority of children’s hospitals in Germany contributing to the network (e.g. 328 participating sites in 2022) [19].

Between October 2019 and September 2022, pediatric hospitals nationwide were requested to report cases of suspected or confirmed *Ureaplasma* CNS infection to the ESPED. Data were collected and anonymized by the ESPED and assessed by the authors. A retrospective study, including patients treated for *Ureaplasma* meningitis in the authors’ own center between 08/2013 and 07/2018, was added to the ESPED-based study. The study was approved by the ethics committee of the University of Würzburg, Germany (#106/19).

### Study population

The ESPED study collected data of preterm and term neonates and infants below 12 months of age with suspected and/or confirmed *Ureaplasma* CNS infection. In our retrospective cohort study adding to this study, we included infants with a history of preterm birth and confirmed *Ureaplasma* CNS infection.

### Definition of *Ureaplasma* CNS infection

Confirmed *Ureaplasma* CNS infection was defined as presence of *Ureaplasma* isolates in cerebrospinal fluid (CSF) and persistent clinical or laboratory signs of meningitis. Suspected *Ureaplasma* CNS infection was defined as hydrocephalus of unknown origin and persistence of pathologic CSF inflammatory markers.

The presence of *Ureaplasma* in CSF was defined as detection by culture method and/or PCR. Both could be conducted at the individual hospitals. Furthermore, as part of the ESPED study, free PCR diagnostics were offered by a central and specialized laboratory at the Institute of Medical Microbiology and Hospital Hygiene at the University Hospital Duesseldorf, Germany.

Normal CSF values were defined as CSF leukocytes of < 20 cells/µL, CSF protein concentrations of < 150 mg/dL, and CSF glucose levels of < 30 mg/dL according to the assessment of CSF parameters in a large study of neonatal meningitis as well as a recent systematic review on normal values for cerebrospinal fluid in neonates [20, 21].

According to current literature, clinical signs of *Ureaplasma* meningitis were acknowledged to be highly variable and were defined as both acute, sepsis-like symptoms (e.g. fever, increased frequency of apneas, irritability, seizures), and chronic presentations with merely discreet and unspecific clinical symptoms including developmental delay as well as the development of internal hydrocephalus not unequivocally associated with intraventricular hemorrhage (IVH) [8, 18]. Internal hydrocephalus is a condition characterized by accumulation of CSF within the brain, resulting in ventricular dilatation.

### Definition of clinical parameters

IVH was classified according to the definition by the German Society of Ultrasound in Medicine (DEGUM) [22]. IVH ≤ °II was defined as low-grade, IVH °III ± periventricular hemorrhagic infarction (PVHI) was defined as high-grade. Respiratory distress syndrome (RDS) was classified according to clinical assessment of work of breathing and inspired oxygen requirement as well as findings upon lung ultrasound and/or X-ray change if available [23]. Clinical or culture-proven sepsis was classified as early-onset sepsis (EOS) if present within the first 72 h of life, and defined as late-onset sepsis (LOS) if occurring after 72 h, according to the criteria of the German national nosocomial infection surveillance system in preterm infants (NEO-KISS) [24]. Retinopathy of prematurity (ROP) was defined according to the International Classification of Retinopathy of Prematurity consensus statement and classified for disease severity and treatment requirement according to the German National Screening and Treatment Guidelines [25, 26]. Bronchopulmonary dysplasia (BPD) was defined as proposed by Jobe and Bancalari [27]. Neurodevelopmental outcomes were assessed at the individual sites by means of neuropediatric follow up examinations using age-specific testing including Hammersmith Infant Neurological Examination, Bayley Scales of Infant Development, and the Wechsler Preschool and Primary Scale of Intelligence. The ESPED survey specified neither the schedule for follow up visits for neurodevelopmental assessment nor the exact assessment tool.

### Statistical analyses

Descriptive statistics include means and ranges to describe quantitative variables where appropriate. Analyses were performed with Microsoft Office Excel 2016 (Microsoft Corporation, Redmont, WA, USA). Missing data was excluded from analysis.

## Results

### Response rate and evaluated cases

During the observation period of three years (2019–2022), five patients meeting the inclusion criteria were reported to the ESPED (patients 1–5, Tables 1, 2 and 3). *Ureaplasma* meningitis was confirmed in all of these patients. No additional cases of suspected, but ultimately not confirmable *Ureaplasma* meningitis were reported.

Three additional patients treated for chronic *Ureaplasma* meningitis in our own center between 2013 and 2018 were included as part of a retrospective analysis (patients 6–8, Tables 1, 2 and 3). All three patients were referred to our hospital from external units with a history of preterm birth and delayed but continuous progression of ventricular dilatation and persistently pathologic CSF inflammatory markers despite multiple courses of empiric antibiotic treatment.

### Demographics, perinatal characteristics, and comorbidities of infants with *Ureaplasma* meningitis

Four infants included in this study were female and four were male. All patients were extremely low birth weight (ELBW) infants with a birth weight range between 600 and 940 g and a gestational age between 24 + 0 and 26 + 3 weeks (Table 1).

Perinatal data were available for 7/8 infants (Table 1). A preterm premature rupture of membranes (PPROM) was reported for six pregnancies. APGAR ranges at 5 and 10 min were 6–8 and 7–10, respectively, and umbilical arterial pH varied between 7,22 and 7,47.

RDS was reported for all infants (8/8) (Table 1). Five out of eight infants were diagnosed with neonatal sepsis, comprising three cases with EOS, one case with LOS, and one infant with a history of both EOS and LOS (Table 1). Two infants developed necrotizing enterocolitis (NEC) requiring surgical treatment. ROP was reported for 6/8 infants, with stage 2 ROP in 3/6 and stage 3 ROP in 3/6 cases. Five infants developed BPD (two mild, three moderate), two infants had no BPD, and data regarding BPD were unavailable for one infant. A patent ductus arteriosus (PDA) requiring therapy was found in 5/8 infants (Table 1). Seven out of eight infants had an IVH ≥ °II. Grade III IVH was present in six infants, uni- or bilateral PVHI occurred in five of these (Table 1).

**Table 1:** Demographics, perinatal characteristics and history of comorbidities of infants with *Ureaplasma* meningitis

No.	Gen-der	GA (weeks)	Birth weight (g)	PPROM	APGAR	UAPh	IVH (grade)	RDS (stage)	BPD	Sepsis	NEC (therapy)	ROP (grade)	Other comor-bidities
1	f	25 + 4	740	yes	5/6/9	7.22	yes (R °III + PVHI)	yes (°II)	mild	LOS	no	yes (°II)	PDA, CDH
2	m	25 + 2	800	yes	5/6/7	7.28	yes (R °III / L °III + PVHI)	yes (n.a.)	mod.	EOS	no	yes (°II)	PDA
3	m	25 + 3	770	yes	4/7/7	7.32	yes (R °II / L °III + PVHI)	yes (°III)	no	EOS	yes (surgery)	yes (°III)	-
4	f	24 + 0	620	yes	6/7/7	7.47	yes (R °III + PVHI / L °II)	yes (n.a.)	mod.	no	no	yes (°II)	MI + per-foration
5	m	24 + 3	600	no	5/6/8	7.42	yes (R °III / L °III)	yes (°II)	mod.	no	no	yes (°III)	PDA
6	f	25 + 3	810	yes	7/8/10	n.a.	yes (R °II / L °II)	yes (°III-IV)	mild	EOS, LOS	no	yes (°III)	PDA
7	m	25 + 3	640	n.a.	n.a.	n.a.	yes (R °II / L °III + PVHI)	yes (°III-IV)	n.a.	n.a.	yes (surgery)	no	n.a.
8	f	26 + 3	950	yes	7/8/8	7.35	no	yes (°III)	no	EOS	no	no	PDA

BPD: bronchopulmonary dysplasia; CDH: congenital diaphragmatic hernia; EOS: early-onset sepsis; GA: gestational age; IVH: intraventricular hemorrhage; LOS: late-onset sepsis; MI: meconium ileus; n.a.: not available; NEC: necrotizing enterocolitis; PDA: patent ductus arteriosus; PPROM preterm premature rupture of membranes; PVHI: periventricular hemorrhagic infarction; RDS: respiratory distress syndrome; ROP: retinopathy of prematurity; UApH: umbilical artery pH

### Signs, symptoms, and diagnosis of *Ureaplasma* meningitis

The onset of clinical symptoms of *Ureaplasma* meningitis varied significantly, beginning in the 2nd week of life in some cases, while gradually developing over a period of months in others (Table 2). An interval of 3 to > 200 days was reported between first signs and diagnosis (Table 2).

Symptoms were mostly non-specific and variable (Table 2). The most commonly reported symptoms were seizures (6/8), prominent fontanelle (5/8), and apnea (4/8). Irritability and refusal to drink were observed in three infants. Fever as a clinical sign was rare and reported in only one patient presenting with acute febrile meningitis (Table 2, infant 7). In all cases, a continuous deterioration of symptoms was noted. All infants were diagnosed with internal hydrocephalus (Table 2). In six infants, internal hydrocephalus developed after a preceding IVH. However, a two-phase course was common in these infants, with delayed progression of hydrocephalus long after IVH resolution. In one case, ventricular dilatation developed > 2 months after IVH diagnosis, and one infant was diagnosed with hydrocephalus without a history of IVH. In all patients, delayed progression of internal hydrocephalus prompted further diagnostics.

Biomarkers of systemic infection were mildly increased or absent in most cases, with C-reactive protein (CRP) levels ranging between 0.42 and 8.5 mg/dL and white blood cell counts between 8,620 and 25,930/µL (Table 2). CSF analysis revealed moderate pleocytosis (16–297/µL) with variable differential count (Table 2). CSF protein levels were significantly increased in most cases (121-4,543 mg/dL). Noteworthy, in all infants, glucose levels were strongly reduced (ranging from 29 mg/dL to not detectable) (Table 2). Uncommonly long persistence of abnormal CSF values combined with progressive ventricular dilatation ultimately prompted *Ureaplasma*-specific microbiological diagnostics.

*Ureaplasma spp.* were detected in the CSF of all eight infants enrolled in this study (Table 3). The identified subtype was *U. parvum* in six and *U. urealyticum* in two cases. Pathogens were identified by both PCR and cultural methods in two cases, by PCR alone in five cases, and solely by culture in one case. Unfortunately, culture-based diagnostics at the individual sites using ready-to-use small volume culture preparations of 10B broth did not allow further culture of isolates and subsequent genotyping or sequencing.

**Table 2:** Clinical presentation and laboratory findings associated with *Ureaplasma*-driven CNS inflammation

No.	Clinical findings		Age at meningitis diagnosis^#^ (days)	Blood parameters			CSF parameters*		
	Symptom	Age at onset (days)		CRP (mg/dL)	Leukocytes (/µL)	Platelets (/µL)	Leukocytes (/µL)	Protein (mg/dL)	Glucose (mg/dL)
1	Hydrocephalus	11	84	0.54	11,300	366,000	16 (differential count n.a.)	4,543	3
	Progression of Hydrocephalus	69							
	Seizures, apneas	78							
	Developmental delay	chronic							
2	Hydrocephalus	< 20	25	1.6	25,930	113,000	297* (differential count n.a.)	1,032*	12*
	Prominent fontanel	< 20							
	Seizures, apneas, temperature instability, irritability	< 20							
3	Hydrocephalus	5	40	2.85	12,900	26,000	43 (neutrophils 45%, lymphocytes 48%)	269	< 2
	Prominent fontanel	10–11							
	Seizures, temperature instability, irritability	10–11							
	Developmental delay	chronic							
4	Hydrocephalus	8	72	1.6	11,000	78,000	76 (differential count n.a.)	409	29
	Prominent fontanel	69							
	Seizures, apneas	69							
	Developmental delay	chronic							
5	Hydrocephalus	10	43	0.78	11,700	190,000	196 (neutrophils 38%, lymphocytes 54%)	730	8
	Developmental delay	chronic							
6	Seizures	36	112	0	8,620	449,000	25 (neutrophils 0%, lymphocytes 93%, monocytes 6%)	347	1
	Hydrocephalus	64							
	Progression of hydrocephalus	93							
	Irritability	93							
	Prominent fontanel	107							
	Developmental delay	chronic							
7	Hydrocephalus	< 14	472	8.5	13,400	407,000	88 (neutrophils 2%, lymphocytes 95%, monocytes 3%)	121	7
	Progression of hydrocephalus	gradual							
	Fever	470							
	Seizures, developmental delay	chronic							
8	Hydrocephalus	28	248	0.42	9,680	525,000	125 (neutrophils 37%, lymphocytes 45%, monocytes 12%)	565	2
	Progression of hydrocephalus	gradual							
	Developmental delay	chronic							

Due to often non-specific symptoms, the exact onset of CNS inflammation could not be determined in all infants. CRP: C-reactive protein; CSF: cerebrospinal fluid, n.a.: not available. *Parameters from first lumbar puncture on d23. ^#^Age at meningitis diagnosis correlates with the age when *Ureaplasma* were first detected in CSF

### Therapy and outcome

Various therapeutic regimens were employed (Table 3). Seven out of eight infants received macrolide therapy, comprising azithromycin monotherapy in two infants, azithromycin + ciprofloxacin in three cases, and azithromycin + chloramphenicol in one case. Erythromycin was part of the therapeutic regimen in one patient. Two infants were initially treated with clarithromycin, but therapy was modified in both cases due to insufficient response (Table 3, infants 3 and 8). Chloramphenicol and doxycyclin were used for monotherapy in one infant each. Pathogen eradication was confirmed by negative PCR and / or sterile CSF culture in seven cases. In one infant, continuously normalized CSF parameters were interpreted as treatment response.

All infants received a ventriculoperitoneal (VP) shunt due to progressive hydrocephalus. At the age of 5–9 months, all eight infants showed developmental delay of varying severity. Additional long-term follow-up data were available for four infants. Three of these infants showed persistently impaired neurocognitive development and were diagnosed with structural epilepsy. Only one patient presented with an age-appropriate development at follow-up assessments at the age of 3 and 6 years.

**Table 3:** Diagnostic, treatment and outcome of preterm infants with *Ureaplasma* meningitis

No.	Pathogen	Detection method	Treatment (duration)	Treatment response	VP shunt	Neurodevelopment^#^
1	*U. parvum*	culture, PCR	Azithromycin i.v. (14 d)	PCR and culture neg.	yes	Developmental delay and structural epilepsy at the age of 26 months
2	*U. parvum*	PCR	Azithromycin i.v. + p.o. (43 d)	CSF parameters improved, culture neg.	yes	Severe combined developmental delay, structural epilepsy, hearing impairment with 30 months
3	*U. urealyticum*	culture	1) Clarithromycin p.o. (14 d) 2) Azithromycin i.v. (5 d) + Ciprofloxacin i.v. (10 d)*	PCR neg.	yes	Developmental delay at the age of 5 months
4	*U. urealyticum*	PCR	Doxycyclin i.v. (14 d)	CSF parameters improved	yes	Developmental delay, seizures at the age of 7 months
5	*U. parvum*	PCR	1) Erythromycin p.o. 2) Chloramphenicol i.v. (23 d) 3) Azithromycin p.o. °	PCR or culture neg.	yes	Developmental delay at the age of 9 months
6	*U. parvum*	PCR	Azithromycin + Ciprofloxacin i.v. (28 d)	PCR and culture neg.	yes	Developmental delay at the age of 9 months
7	*U. parvum*	PCR	Azithromycin + Ciprofloxacin i.v. (21 d)	PCR and culture neg.	yes	Developmental delay at the age of 21 months, structural epilepsy, cerebral palsy
8	*U. parvum*	culture, PCR	1.) Clarithromycin i.v. (3 d) 2.) Chloramphenicol i.v. (21 d)*	PCR and culture neg.	yes	Minor developmental delay at the age of 21 months, appropriate at the age of 3 and 6 years

i.v.: intravenous; p.o.: peroral; U.: *Ureaplasma*; VP: ventriculoperitoneal. *Consecutive antibiotic regimens; °Exact therapeutic regimen not available. ^#^As assessed at the individual site by means of neuropediatric follow up examinations using age-specific testing including Hammersmith Infant Neurological Examination, the second or third edition of the Bayley Scales of Infant Development and the Wechsler Preschool and Primary Scale of Intelligence

## Discussion

*Ureaplasma* spp. are widely accepted as pathogens associated with chorioamnionitis and preterm birth, but their role in neonatal morbidity is still discussed controversially [28]. Against the background of several case reports describing neonatal meningitis and CNS inflammation caused by *Ureaplasma* spp [8], this is the first study systematically assessing the incidence of *Ureaplasma* meningitis in neonates, choosing a nationwide surveillance approach. With a total of eight preterm infants included in the final analysis, this study provides the largest series of cases of neonatal *Ureaplasma* meningitis published to date, offering novel insights into the clinical picture as well as therapeutic challenges.

Our results show that clinical symptoms of *Ureaplasma*-driven CNS inflammation may be variable, non-specific, and not clearly distinguishable from other comorbidities. Some individuals in this study became apparent with acute, sepsis-like symptoms, fever or temperature instability, apnea, and seizures (Table 2). Similar symptoms were documented in previous case reports [8]. Other infants in our cohort, however, presented with a gradual progression of neurological symptoms, such as developmental delay or progression of hydrocephalus, rather than acute signs of infection.

In fact, the presence of internal hydrocephalus was one of the most consistent findings and was reported for all infants included in this study. Again, this is in accordance with previous literature, describing the occurrence of hydrocephalus in the majority of *Ureaplasma* meningitis cases [8]. However, it is important to emphasize that all but one infant had been diagnosed with IVH (Table 1) prior to diagnosis of *Ureaplasma* CNS infection. CSF accumulation causing internal hydrocephalus commonly results from obstruction in CSF circulatory pathways following either IVH or other acquired abnormalities including meningitis. Thus, we cannot clearly differentiate between post-hemorrhagic and *Ureaplasma*-driven hydrocephalus in these infants. Yet, ventricular dilatation was also observed in one infant with low grade IVH and in the neonate without IVH. Notably, all infants with a history of intracranial hemorrhage experienced delayed progression of hydrocephalus, partly weeks or even months after IVH manifestation– eventually prompting lumbar puncture. Thereafter, an uncommonly long persistence of high and very high CSF protein levels, potentially reflecting barrier disruption and inflammation rather than hemorrhage, prompted further diagnostics, and ultimately, *Ureaplasma* isolates were detected.

While IVH and the associated blood brain barrier (BBB) affection may have facilitated *Ureaplasma* invasion into the CNS, vice versa, *Ureaplasma* spp. may have promoted IVH in our cases. No doubt, IVH is a common condition in preterm neonates, with an incidence of about 20–25% in very low birth weight infants, again affecting primarily the most immature infants [29]. However, inflammation may promote development of IVH, and as such, *Ureaplasma* spp. have been discussed as potentially contributing factor [18, 28]. Clinical studies regarding a causal relationship between *Ureaplasma* spp. and IVH are scarce and contradictory, with some authors describing a higher risk for IVH development in *Ureaplasma*-exposed preterm infants, whereas others did not confirm any correlation [18, 30–33]. Animal studies on preterm IVH provide evidence of BBB disruption upon hemorrhage [34]. At the same time, *Ureaplasma* spp. themselves may compromise BBB integrity. This hypothesis is strengthened by experimental data deriving from animal models and in vitro studies in human brain microvascular endothelial cells (HBMEC), describing potentially underlying mechanisms [13–17]. Animal models indicate *Ureaplasma*-driven CNS inflammation and structural brain injury [14, 35] that may ultimately drive inflammation-associated IVH. In vitro studies investigating the potential role of *Ureaplasma* spp. in neonatal meningitis describe induction of apoptosis-related caspases and modulation of BBB receptors, such as the atypical chemokine receptor 3 (ACKR3) and the C-X-C chemokine receptor type 4 (CXCR4) in *Ureaplasma*-exposed HBMEC [13, 15–17]. One may hypothesize that particularly the coincidence of IVH and systemic *Ureaplasma* infection may disrupt the BBB, allow pathogen entry into the CNS, and predispose for the consecutive development of meningitis.

All individuals in our study were former ELBW infants. These findings are in accordance with data from previous case reports, indicating an inverse relation of *Ureaplasma*-associated CNS inflammation and gestational age with affection of primarily the most immature infants [8–10]. Yet, single cases of CNS infection caused by *Ureaplasma* spp. were described in term neonates or immunocompromised adults [8, 36, 37]. As opposed to term neonates, extremely immature preterm infants appear to be particularly prone to subacute and chronic *Ureaplasma*-driven CNS infection [8]. Noteworthy, in some cases, neurological symptoms preceded the diagnosis for several months. Numerous reasons may account for the increased susceptibility of very immature preterm infants: *Ureaplasma* transmission from mother to infant is inversely related to gestational age, with highest transmission rates in the most immature infants [3]. Previous studies revealed *Ureaplasma* detection rates of almost 20% in cord or early venous blood samples of prematurely born infants [3, 31]. Particularities of preterm immune responses, such as cytokine imbalance and reduced complement function, might, in case of blood stream invasion, impair pathogen eradication and promote sustained infection [28, 38, 39]. Upon bacteremia, the immature BBB appears to frequently allow passage of *Ureaplasma* spp. into the CNS. It is worth mentioning that *Ureaplasma* spp. were detected in CSF samples of 19% of preterm infants with a birth weight ≤ 1500 g undergoing lumbar puncture for sepsis work-up [31].

PPROM was reported for six out of eight infants in our study. PPROM is a major risk factor for ascending infection in pregnant women vaginally colonized with *Ureaplasma* spp. and may increase the risk of pathogen transmission to the offspring [40]. The high frequency of PPROM in our cohort might confirm rupture of membranes as a relevant risk factor for perinatal *Ureaplasma* exposure and, potentially, *Ureaplasma*-associated adverse neonatal outcome including meningitis. Noteworthy, prenatal pathogen transmission is not confined to the event of PPROM, since presence of *Ureaplasma* in blood or CSF of preterm infants was described even in pregnancies with a history of membrane rupture < 1 h [31].

Equaling variations in clinical manifestation, systemic inflammatory marker responses were heterogenous. Notably, we observed signs of systemic inflammation particularly in those infants with acute clinical infectious symptoms but found no measurable responses in other cases. CSF inflammatory parameters, however, were pathologic in all patients enrolled. All study infants showed significantly decreased CSF glucose levels as the most remarkable finding. Furthermore, we often found elevated CSF protein levels accompanied by a remarkably mild CSF pleocytosis and low granulocyte numbers. These findings may indicate barrier disruption rather than fulminant inflammation, and similar CSF parameters were described in previous reports of *Ureaplasma* CNS infection [8]. While pleocytosis, elevated CSF protein levels, and low glucose levels are non-specific and may all be ascribed to CNS infections of other origin or may have been caused by preceding IVH, our study as well as existing literature suggest that hypoglycorrhachia with only mild or moderate leukocytosis is a pathognomonic finding of *Ureaplasma* meningitis and might stipulate targeted diagnostics [8, 9].

Once *Ureaplasma* meningitis is suspected, specific microbiological CSF diagnostics should be initiated. Since standard culture methods are not appropriate for *Ureaplasma* detection, targeted diagnostics using specialized culture media have to be employed, ideally amended by more sensitive PCR assays [41]. In our study, some patients had undergone previous standard microbial CSF diagnostics, which had failed to identify a pathogen. We observed a latency of up to 200 days and more between the onset of symptoms and the diagnosis of *Ureaplasma* meningitis. However, in some cases, targeted diagnostics had allowed pathogen detection within only a few days. Certainly, early diagnostics is invaluable for early initiation of adequate anti-infective and, at best, anti-inflammatory treatment, limiting both infection and inflammation-induced CNS injury.

Treatment of neonatal CNS infection with *Ureaplasma* spp. is challenging, and therapeutic regimens regarding antibiotics, dosage, and duration are undefined [8]. While antibiotics affecting bacterial cell walls are ineffective in *Ureaplasma* therapy, macrolides, tetracyclines, clindamycin, chloramphenicol, and quinolones constitute potent drugs [42]. However, tissue penetration of individual antibiotics as well as potential side effects in neonates have to be taken into critical consideration. Potential side effects of macrolides mainly include QT-interval prolongation and an increased risk of developing infantile hypertrophic pyloric stenosis. As far as the off-label use of chloramphenicol is concerned, infants need to be closely monitored for potential hematologic side effects including anemia, leukopenia and thrombocytopenia. Moreover, the gray baby syndrome is a rare but serious and often fatal condition associated with high serum levels of chloramphenicol.

The wide ranges of off-label therapy regimens used in the infants enrolled in this study illustrates the need for more research in this field and the necessity of future therapeutic guidelines. As far as *Ureaplasma*-associated adverse pregnancy outcomes and the development of BPD are concerned, the macrolide azithromycin has attracted interest as an apparently safe and effective antimicrobial agent over the years [43, 44]. To date, only two previous case reports have described the use of azithromycin as part of the therapeutic regimen in neonatal *Ureaplasma* meningitis [12, 45]. While macrolides generally show a poor CNS penetration, azithromycin has been successfully used in a number of cases assessed in this study, potentially due to inflammation-associated affection of BBB integrity. To the best of our knowledge, we herein present the first two cases of successful azithromycin monotherapy of *Ureaplasma* meningitis, with verified pathogen eradication and improved CSF parameters in both infants. It remains to be elucidated if this therapeutic strategy proves successful in a larger number of *Ureaplasma* meningitis patients. Also, appropriate dosing and duration of such treatment would need to be assessed.

Previous literature indicates that developmental impairment is a common sequela of neonatal *Ureaplasma* meningitis [8]. Despite pathogen eradication, unfortunately, all patients included in the current study were diagnosed with developmental delay at discharge. Data on long-term follow-up was available for four infants. Notably, three out of these four infants presented with severe cognitive impairment, including developmental delay and structural epilepsy, on long-term follow-up. This may be due to either IVH, meningitis, hydrocephalus, or a combination of these disease entities. Noteworthy, an association between adverse neurocognitive and motor outcome at 2 years corrected age and prenatal *Ureaplasma* exposure of preterm infants has been reported previously [33, 46]. This commonly described long-term neurocognitive impairment indicates a relevant role of *Ureaplasma*-driven CNS inflammation in neonatal medicine despite apparently low total numbers.

Since all reported cases were ultimately confirmed as *Ureaplasma* meningitis, this study lacks a control group, which would have helped to better estimate the prevalence of *Ureaplasma*-driven neuroinflammation. This study is furthermore limited by its small sample size. Despite our nationwide surveillance approach, only five patients were reported in the 3-year study period. This may indicate a very low incidence of *Ureaplasma* meningitis in neonates. Yet, the herein presented numbers might be of considerable relevance in the cohort of ELBW infants, comprising about 0.6–0.7% of all births in Germany [47]. Moreover, the true incidence of *Ureaplasma*-driven neuroinflammation is likely to be distinctly higher. Given the often non-specific clinical presentation as well as the necessity of targeted microbiological diagnostics, and considering *Ureaplasma* detection rates of up to 19% in CSF samples of preterm infants in previous studies [31], we hypothesize that a relevant number of *Ureaplasma* associated meningitis cases remains undiagnosed. Our results call for larger studies systematically assessing *Ureaplasma* colonization and clinical outcome in preterm infants, including neonatal CNS infection and potentially associated brain injury.

## Conclusion

This is the first study systematically assessing *Ureaplasma* meningitis in neonates. Our results provide important insights in *Ureaplasma*-driven neonatal neuroinflammation. The most immature preterm infants reflect the group at highest risk. While clinical symptoms and laboratory findings can be inconclusive, internal hydrocephalus and low CSF glucose levels appear to be indicators of neonatal *Ureaplasma* meningitis. If *Ureaplasma* meningitis is suspected, targeted CSF diagnostics need to include adequate cultural methods as well as *Ureaplasma* PCR. While azithromycin has successfully been used in individual cases with neonatal *Ureaplasma* meningitis, most antibiotic regimens are off-label in preterm infants, and therapeutic recommendations are pending. Severe impact on short- and long-term outcome was observed in all infants with *Ureaplasma* meningitis in this study, highlighting the relevance of invasive *Ureaplasma* CNS infection for the individual despite being seemingly rare in total numbers. However, due to non-specific symptoms and challenging diagnostics, a relevant number of cases might remain undiagnosed so far. *Ureaplasma* meningitis should always be taken into consideration in preterm infants, particularly very immature ones, presenting with progressive ventricular dilatation paralleled by clinical and CSF signs of CNS inflammation.

## Data Availability

Data is provided within the manuscript.
